# A eukaryotic-type signalling system of *Pseudomonas aeruginosa *contributes to oxidative stress resistance, intracellular survival and virulence

**DOI:** 10.1186/1471-2164-12-437

**Published:** 2011-08-31

**Authors:** Jana Goldová, Aleš Ulrych, Kamil Hercík, Pavel Branny

**Affiliations:** 1 Cell and Molecular Microbiology Division, Institute of Microbiology of the ASCR, v.v.i., Academy of Sciences of the Czech Republic, Vídeňská 1083, 142 20 Prague 4, Czech Republic; 2 Laboratory of Molecular Genetics, National Institute of Child Health and Human Development, National Institutes of Health, Bethesda, MD 20892, USA

## Abstract

**Background:**

The genome of *Pseudomonas aeruginosa *contains at least three genes encoding eukaryotic-type Ser/Thr protein kinases, one of which, *ppkA*, has been implicated in *P. aeruginosa *virulence. Together with the adjacent *pppA *phosphatase gene, they belong to the type VI secretion system (H1-T6SS) locus, which is important for bacterial pathogenesis. To determine the biological function of this protein pair, we prepared a *pppA-ppkA *double mutant and characterised its phenotype and transcriptomic profiles.

**Results:**

Phenotypic studies revealed that the mutant grew slower than the wild-type strain in minimal media and exhibited reduced secretion of pyoverdine. In addition, the mutant had altered sensitivity to oxidative and hyperosmotic stress conditions. Consequently, mutant cells had an impaired ability to survive in murine macrophages and an attenuated virulence in the plant model of infection. Whole-genome transcriptome analysis revealed that *pppA-ppkA *deletion affects the expression of oxidative stress-responsive genes, stationary phase σ-factor RpoS-regulated genes, and quorum-sensing regulons. The transcriptome of the *pppA-ppkA *mutant was also analysed under conditions of oxidative stress and showed an impaired response to the stress, manifested by a weaker induction of stress adaptation genes as well as the genes of the SOS regulon. In addition, expression of either RpoS-regulated genes or quorum-sensing-dependent genes was also affected. Complementation analysis confirmed that the transcription levels of the differentially expressed genes were specifically restored when the *pppA *and *ppkA *genes were expressed ectopically.

**Conclusions:**

Our results suggest that in addition to its crucial role in controlling the activity of *P. aeruginosa *H1-T6SS at the post-translational level, the PppA-PpkA pair also affects the transcription of stress-responsive genes. Based on these data, it is likely that the reduced virulence of the mutant strain results from an impaired ability to survive in the host due to the limited response to stress conditions.

## Background

*Pseudomonas aeruginosa *is a gram-negative, opportunistic pathogen that causes serious acute and chronic infections and presents considerable complications for burned patients, patients with cystic fibrosis, and those in other immunocompromised states [1]. The production of a wide variety of virulence factors that enhance the survival of the bacterium within the host is regulated via density-dependent quorum sensing and by the growth phase-dependent RpoS system [2]. Moreover, *P. aeruginosa *has an intrinsic ability to resist a variety of antimicrobial agents. This ability is further strengthened within the infected host by the formation of highly antibiotic-resistant biofilms [3,4]. These features make the treatment of *P. aeruginosa *infections very difficult and, therefore, the identification of new therapeutic targets has become very important.

Protein phosphorylation is considered the universal language for inter- and intra-cellular communication in all living organisms. This process, catalysed by protein kinases, enables the translation of extracellular signals into cellular responses and also allows for adaptation to a constantly changing environment. In recent years, a number of bacterial eukaryotic-type Ser/Thr protein kinases (STPKs) and phosphoprotein phosphatases have been identified. These enzymes operate in many bacteria, in which they constitute a signalling network that is independent of the canonical two-component system circuits.

Prokaryotic STPKs regulate various cellular functions, such as developmental processes [5,6], primary and secondary metabolism [7,8], stress responses [9], and biofilm formation [10].

STPKs also play a role in the virulence of many bacterial pathogens such as streptococci, *Staphylococcus aureus *[11], *Mycobacterium tuberculosis *[12], *Yersinia *spp. [13,14], and *P. aeruginosa *[15-17].

It has been observed that the protein kinase PpkA of *P. aeruginosa *characterized by Wang et al. [17] and Motley and Lory [18] is specifically and highly induced during the infection of neutropenic mice and, therefore, it has been implicated in *P. aeruginosa *virulence [17]. The *ppkA *gene has also been identified as a component of the HSI-I genetic locus that encodes a novel secretion system (H1-T6SS), designated as the type VI secretion system (T6SS), which functions in bacterial pathogenesis [19]. It has been shown that the secretion of Hcp1 protein, VgrG proteins and Tse1-3 proteins is post-translationally regulated by PpkA [15]. Hcp1 interacts with VgrG proteins VgrG1 and VgrG4 [15,20]. The function of the Hcp/Vgr complex remains unclear; however, it is believed that the proteins are extracellular structural components of the secretion apparatus [15,21]. Third group of proteins secreted by H1-T6SS is represented by Tse1-3, where Tse2 is the toxin component of toxin-immunity system and is specifically targeted to bacteria [15]. Phosphorylation of the Fha1 protein containing the forkhead-associated (FHA) domain by PpkA leads to the recruitment of T6SS components to the inner membrane, which results in the secretion of periplasmic Hcp1 and other proteins across the outer membrane. In addition, PpkA activity is antagonised by its cognate phosphatase, PppA, which exhibits phosphatase activity on phosphorylated Fha1. Protein TagR, whose gene is downstream of *pppA-ppkA*, is required for PpkA activation [21].

In the present study, we have carried out phenotypic investigations of a *pppA-ppkA *double mutant. The *pppA-ppkA *null mutant showed decreased resistance to H_2_O_2_-induced oxidative stress and increased sensitivity to macrophage-mediated killing. In addition, mutations in these genes led to a lower sensitivity to osmotic stress. These results suggest that the expression of the PppA-PpkA regulatory pair can contribute to *P. aeruginosa *resistance to diverse environmental cues. Furthermore, we show that the *pppA-ppkA *mutation affects *P. aeruginosa *virulence in the lettuce leaf model of infection.

Microarray analysis was used to analyse the transcriptomes of the mutant and wild-type strains under standard and oxidative stress conditions. This analysis revealed that the *pppA-ppkA *mutation is pleiotropic. Several functional gene categories have been identified that could account for a reduced stress response and bacterial fitness. In addition to the oxidative stress-responsive genes, PppA-PpkA affects the expression of genes regulated by stationary phase σ-factor RpoS as well as the *las *and *rhl *quorum sensing regulons. When exposed to hydrogen peroxide stress, the *pppA-ppkA *mutant exhibited an impaired response of the stress-induced genes and altered expression of genes of the quorum sensing (QS)-regulated PA2134-2192 locus and Pho regulon genes.

Collectively, the results led us to speculate that the PppA-PpkA pair can sense external stress signals and regulate the response of *P. aeruginosa *to environmental conditions through its possible connection with RpoS/QS regulons.

## Methods

### Bacterial strains, plasmids, and growth conditions

The bacterial strains and plasmids used in this study are listed in Table 1. *Escherichia coli *JM109 was grown in Luria-Bertani (LB) medium at 37°C. *P. aeruginosa *PAO1 was grown in LB medium or minimal M9 medium if not otherwise stated. M9 medium contained 2% glucose as a carbon source in all cases. Only for growth characteristics comparison glycerol (2%), instead of glucose as a carbon source, was used. Elastin broth plates [22] and calcium caseinate agar [23] were used to determine elastase activity. SW blue plates [24] were used for rhamnolipid production. Haemolytic activity was determined on blood agar. Antibiotics were added when necessary at the following concentrations (in μg•ml^-1^): ampicillin (Ap), 100; gentamicin (Gm), 15 (for *E. coli*); carbenicillin (Cb), 250; gentamicin, 100 (for *P. aeruginosa*).

**Table 1:** Bacterial strains and plasmids used in this study.

Strain/plasmid	Genotype or description^a^	Source
**Strains**		
***P. aeruginosa***		

PAO1	wild type	T. Köhler
Δ*pppA-ppkA*	PAO1 with unmarked deletion of *pppA-ppkA *	This work
PAO1::tn7TLAC	PAO1::mini-tn7T-LAC	This work
Δ::tn7TLAC	Δ*pppA-ppkA*:: mini-tn7T-LAC	This work
Δ::tn7TLACpak	Δ*pppA-ppkA*:: mini-tn7T-LAC-*pppA-ppkA*	This work

***E. coli***		

XL1-blue	*rec*A1 *end*A1 *gyr*A96 *thi hsd*R17(r_k_^-^, m_k_^+^) *sup*E44 *rel*A1 lac [F' *pro*AB^+ ^*lac*Iq Δ(*lac*Z)M15::Tn10]	Stratagene
JM109	*end*A1 *rec*A1 *gyr*A96 *thi hsd*R17 (rk^-^mk^+^) *sup*E44 Δ(*lac-pro*AB), [F' *tra*D36 *pro*AB *lac*Iq Δ(*lac*Z)M15]	Promega

**Plasmids**		

pEX18Ap	Ap^R^, *ori*T^+ ^*sac*B^+^, gene repacement vector with MSC from pUC18	(24)
pPS858	Ap^R^, Gm^R^, *gfp*^+^, contains Gm^R^-GFP FRT cassette	(24)
pFLP2	Ap^R^, contains FLP recombinase gene	(24)
pUC18-mini-Tn7T-LAC	Ap^R^, Gm^R^, mini-Tn7T-Gm Gateway-compatible cloning and delivery vector with *lacI^q^-Ptac *expression cassette	(7)
pTNS2	Helper plasmid for integration of the expression casette	(7)
pUC18miniTn7TLACpak	pUC18-mini-Tn7T-LAC expressing *pppA-ppkA*	This work

^a^Ap^R^, resistant to ampicillin; Gm^R^, resistant to gentamicin

### Construction of the *P. aeruginosa *Δ*pppA*-*ppkA *mutant

The deletion was achieved by crossover PCR mutagenesis [25] and an improved method for gene replacement in *P. aeruginosa *[26]. Crossover PCR was used to generate a fragment that linked 800-bp and 1,035-bp fragments corresponding to downstream and upstream flanking regions of the *pppA-ppkA *loci, respectively. Oligonucleotides used in this study are listed in Additional file 1. The final construct was prepared by the directional cloning of fragments into a suicide vector, pEX18Ap, and the subsequent cloning of the Gm^R^-GFP FRT cassette from pPS858 into a *Bam*HI site. The resulting plasmid was then electroporated into wild-type *P. aeruginosa *cells. The Gm^R ^marker was removed by Flp-mediated excision following previously described methods [26]. The mutant obtained was verified by both PCR and Southern blot analysis.

### Complementation

For complementation analysis, recombinant plasmids were transformed into the *P. aeruginosa *PAO1 and mutant strains by electroporation. Single-copy complementation was achieved by cloning the DNA fragment into pUC18-mini-Tn7T-LAC, a mini-Tn7 vector containing a *tac *promoter, followed by integration into the single *att*Tn7 site on the *P. aeruginosa *chromosome [27]. Specifically, a 4,116-bp *SacI-KpnI *DNA fragment containing the *pppA-ppkA *genes was amplified by PCR using the PPPAinfpUCF-PPKAinfpUCR oligonucleotide pair. The PCR fragment was subcloned into pUC18-mini-Tn7T-LAC to yield pUC18miniTn7TLACpak, in which the *pppA-ppkA *genes are expressed from the *tac *promoter. The mini-Tn7T-LACpak segment contained on this plasmid was transposed into the chromosome of the Δ*pppA-ppkA *strain by coelectroporation with pTNS2. Insertion events were verified by PCR, and the Gm^R ^marker was removed by Flp-mediated excision following previously described methods [26]. Control PAO1 and Δ*pppA-ppkA *strains contained mini-Tn7TLAC integrated into the chromosome. The strains obtained were verified by both PCR and Southern blot analysis.

### Motility assays

Swarming motility was determined as described by Kohler et al. [24]. All strains were inoculated by toothpick with individual colonies from a fresh LB agar plates. Incubation was done at 37°C for 24 hours. Swimming motility was determined as described by Whitchurch et al. [28]. Cells were stab-inoculated into a LB plate with 0.3% agar. The motility was examined by monitoring the circular turbid zone formed by the bacterial cells migrating away from the point of inoculation. Twitching motility was determined as described by Schweizer and Choi [29]. All strains were stab-inoculated through a LB plate containing 1% agar and grown 72 hour at 37°C. The agar was carefully removed, and residue adhering to the Petri dish was stained with Coomassie brilliant blue R250 (0.05% in 40% methanol, 10% acetic acid).

### Quantification of pigments and elastase assay

Overnight LB cultures were diluted in fresh LB medium and were grown for 24, 48, and 72 h at 37°C. For extraction of pyocyanin, a 5 ml sample of the supernatant was mixed with 5 ml chloroform and the lower organic layer was separated. To this layer, 1.5 ml 0.2 M HCl was added and pyocyanin-rich aqueous phase was separated. The amount of pyocyanin within the extracted layer was determined by measuring the OD_520 _[30] and normalized to the respective cell densities (OD_600_).

Elastase activity was determined in these same cultures from 100 μl of supernatants using Elastin-Congo Red (Sigma) and spectrophotometric measurement at 495 nm as previously described by Rust et al. [31].

Pyoverdine concentrations were calculated from supernatant of cultures grown 24 hours in Casamino Acids medium (CAA) by spectrophotometric measurement at 405 nm [32]. Amount of pyoverdine was normalized to the respective cell densities (OD_600_).

### Quantification of alginate

Selected strains were grown on LB agar (three plates per strain) at 37°C for 48 hours. Cells were washed from plates and resuspended in 0.9% NaCl. Alginate isolation and purification were performed as described by May and Chakrabarty [33]. Amount of alginate was normalized to the respective bacterial dry weight.

### Antibiotic resistance test

For general tests with chloramphenicol, carbenicillin, pefloxacin, tetracycline and trimethoprim, we used gradient antibiotic plates [34]. Minimal inhibitory concentrations (MICs) for chloramphenicol and carbenicillin were determined by population analysis profiling [35]. Briefly, serial dilutions of early stationary phase cultures were plated on LB agar plates containing different concentrations of chloramphenicol or carbenicillin. The plates were incubated at 37°C for 24 h, and the number of bacteria capable of forming colonies in the presence of various antibiotic concentrations was counted.

### Macrophage-mediated bactericidal assay

Macrophage-mediated bactericidal assays were carried out as described previously [36] with slight modification. The immortalised murine macrophage cell line J774.2 was used to examine the rate of survival *in vitro*. Macrophages were grown in Dulbecco's modified Eagle's medium (low glucose) supplemented with 5 mM glutamine and 5% fetal bovine serum (Sigma) in a 5% CO_2 _atmosphere at 37°C. Mid-log-phase *P. aeruginosa *grown in LB broth at 37°C was collected by centrifugation at 5,000 g and resuspended to an OD_600 _of 0.4 in PBS. Macrophages (5 × 10^7^) were incubated in Dulbecco's modified Eagle's medium-5% fetal bovine serum with the wild-type and mutant strain of *P. aeruginosa *(5 × 10^7 ^CFU) for 30 min at 37°C. In order to eliminate extracellular *P. aeruginosa*, three washes by centrifugation at 1,500 g for 5 min at room temperature in Hank's buffered saline solution were performed. After the final wash, macrophages were allowed to adhere to tissue culture flasks in Dulbecco's modified Eagle's medium supplemented with gentamicin (400 mg ml^-1^). Parallel control samples for counting initial number of phagocyted bacteria were washed three times by centrifugation in Hank's buffered saline solution with gentamicin (400 mg•ml^-1^), resuspended in equal volumes of cold sterile water, and lysis was completed by vortexing the macrophages three times for 1 min and incubating the macrophages on ice for 15 min before bacterial viability was assessed by plating. After one hour of incubation macrophages with internalized bacteria were harvested, lyzed as described and bacterial viability was assessed by plating. % of survival represents % of bacteria surviving after internalization.

### *In vitro *stress experiments

The sensitivity of cells to oxidative stress was tested by exposing aliquots of stationary-phase cultures diluted in LB medium (10^7 ^CFU•ml^-1^; OD _600 _= 0.01) at 37°C to 1 mM, 10 mM, 25 mM, 30 mM, 40 mM and 50 mM H_2_O_2 _for 1 h. Viable cells were counted by plating them onto agar plates before and after exposure to H_2_O_2_, and results are expressed as survival percentages.

To study the effect of osmotic stress on the wild-type and mutant strains, stationary-phase cultures diluted in LB medium (10^7•^CFU ml^-1^; OD _600 _= 0.01) were grown overnight in LB containing 1.3, 1.6 or 2 M NaCl at 37°C with aeration, and serial dilutions of the samples were plated on LB plates to determine the CFU. Results are expressed as survival percentages.

### Plant virulence assays

A lettuce leaf virulence assay was performed as described previously, [37]. Briefly, 10 μl of stationary-phase cultures were diluted in LB medium (10^7 ^CFU•ml^-1^; OD _600 _= 0.01), washed and resuspended in 10 mM MgSO_4_. Samples were then inoculated into the midribs of Romaine lettuce leaves. Petri dishes containing Whatman filter paper soaked with 10 mM MgSO_4 _and inoculated leaves were kept in a growth chamber at 28°C for five days. Symptoms were monitored daily.

### RNA isolation and Affymetrix GeneChip microarrays

The wild-type and mutant strains were cultured in minimal M9 medium at 37°C up to early stationary phase (OD_600 _= 0.8). Oxidative stress was generated by treatment of cultures at OD_600 _= 0.8 with 10 mM H_2_O_2 _for 15 minutes. Three independent replicates of total RNA were isolated from each strain using an RNeasy mini-kit with on-column DNase digestion (Qiagen) according to the manufacturer's instructions. For DNA microarrays, two replicates of total RNA (10 μg) from each strain were used for cDNA synthesis, fragmentation, and labelling according to the Affymetrix GeneChip *P. aeruginosa *Genome Array Expression Analysis protocol (Affymetrix, Santa Clara, CA, USA). Briefly, random hexamer primers (final concentration, 25 ng•μl^-1^; Invitrogen) were added to the total RNA (10 μg) along with *in vitro*-transcribed *Bacillus subtilis *control spikes, as described in the Affymetrix GeneChip *P. aeruginosa *Genome Array Expression Analysis protocol. cDNA was synthesised using Superscript II (final concentration of 25 U•μl^-1 ^(Invitrogen)) according to the manufacturer's instructions under the following conditions: 25°C for 10 min, 37°C for 60 min, 42°C for 60 min, and 70°C for 10 min. RNA was removed by alkaline treatment and subsequent neutralisation. The cDNA was purified using a GeneChip Sample Cleanup Module (Affymetrix) and was eluted in 12 μl of EB Buffer. The cDNA was fragmented with DNase I (0.6 U per μg of cDNA; Amersham) at 37°C for 10 min and then end-labelled with biotin-ddUTP using a GeneChip^® ^DNA Labelling Reagent (Affymetrix) at 37°C for 60 min. Proper cDNA fragmentation and biotin labelling were determined by gel mobility shift assay performed with NeutrAvadin (Pierce), followed by electrophoresis through a 4-20% Tris-Borate gel and subsequent DNA staining with SYBR gold (Invitrogen). Fragmented labelled cDNA samples were hybridised to the array and scanned with an Affymetrix GeneChip Scanner 3000 7G.

### Microarray data analysis

Raw data (CEL files) were imported into R statistical software [38] and normalised using the GCRMA method (GC Robust Multi-array Average (GCRMA) background adjustment, quantile normalisation, and median polish summarisation) [39]. The normalised data were tested for differences in expression using the moderated *t*-test from the limma [40] package from the Bioconductor [41] repository. Raw *P *values were corrected using the Benjamini & Hochberg method [42]. Microarray data were deposited in ArrayExpress database with accession number E-MEXP-3117.

### Real-Time PCR

RT-PCRs were carried out in an iCycler^® ^thermal cycler (Bio-Rad). The same RNA samples were used for real-time PCR analysis as those used for microarray analysis together with the third replicates of isolated RNA samples. One microgram of RNA served as a template for cDNA synthesis with ImProm reverse transcriptase (Promega). The cDNA was then used as a template in a PCR performed with a Sybr green I JumpStart *Taq *ReadyMix kit (Sigma). Three biological replicates with three technical replicates each were used for each gene. Changes in gene expression between the wild-type and mutant strain, with *proC *(PA0393, pyrroline-5-carboxylate reductase, prolin metabolism, a housekeeping gene) as a reference [43], were estimated by the ΔΔC_T _method [44,45].

### Statistical analysis

The Student's *t*-test was performed to determine statistical significance between pairs of experimental groups. A *P *value < 0.05 was considered statistically significant. All experiments were repeated at least three independent times.

## Results and discussion

### The Δ*pppA-ppkA *mutant exhibits reduced ability to grow in minimal medium and decreased production of pyoverdine

Prior to studying the effect of the *pppA-ppkA *mutation on the expression profiles, we first examined the *pppA-ppkA *null mutant for the production of principal virulence factors, including LasA and LasB elastase, phospholipase C, rhamnolipids, pyocyanin, and pyoverdin, on dedicated solid media. The mutant strain demonstrated a delayed production of several virulence factors, particularly on the test plates containing glycerol where the growth of the mutant was significantly slower when compared to the wild-type strain PAO1.

**Figure 1:**
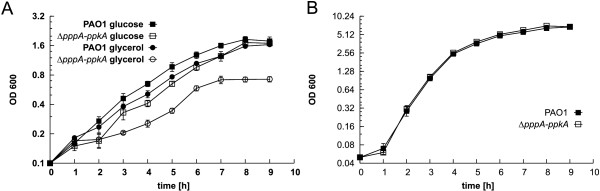
**Characteristics of *Pseudomonas aeruginosa *PAO1 wild-type and Δ*pppA-ppkA *strains**. **(A)**Growth curves in M9 minimal medium with glucose or glycerol as the sole carbon source. **(B) **Growth curves in complex LB medium. The standard errors of the means for three independent experiments are shown. Where error bars are not shown, the standard error was within the size of the symbol.

Therefore, we examined growth characteristics of the wild-type and the mutant strains in both minimal and complex media. In liquid minimal medium M9 with glucose as the sole carbon source at 37°C, the mutant strain had extended lag phase when compared to the parent strain (Figure 1A). In minimal M9 medium with glycerol as the sole carbon source, the mutant strain showed significantly reduced growth rate compared to that of the parent strain (Figure 1A). The calculated doubling time of the mutant grown in the glucose-containing media was 96 (± 7) min compared to 86 (± 6) min for the wild-type strain. The doubling time of the mutant grown in the glycerol-containing media was increased to 120 (± 7) min compared 102 (± 7) min for the parent strain. In addition, cultures of the mutant strain did not achieve the same final optical density as the parent strain in the M9 medium with glycerol. Similar growth defect was also observed in King's A and King's B media containing glycerol as carbon source (Additional file 2). In contrast, the growth characteristics of the mutant were unaltered when it was grown in either complex LB medium with a doubling time of 34 min, (Figure 1B), or CAA medium (Additional file 2). Production of the blue pigment, pyocyanin, in the mutant strain was only slightly decreased when the mutant was grown on solid LB agar (data not shown). No difference in pyocyanin production was observed in liquid LB medium. On the other hand, the mutant strain secreted a reduced amount of pyoverdine (57% of the amount of the parent strain) when both strains were grown in liquid CAA medium (Table 2).

**Table 2:** Production of virulence factors by PAO1 and Δ*pppA*-*ppk*A and MIC profiles

	Strain					
	PAO1			Δ*pppA*-*ppk*A		
	
Virulence factor	24 h	48 h	72 h	24 h	48 h	72 h
elastases - elastine degradation	+	+	+	+	+	+
elastases - casein degradation			+			+
pyocyanin	+	+	+	+	+	+
pyoverdine (OD_405_/OD_600_)^a^	0.68 (± 0.032)			0.39 (± 0.054)		
phospholipase C	+			+		
swarming motility	+			+		
swimming motility	+			+		
twitching motility			+			+
alginate		+			+	

**MIC (μg•ml^-1^)^b^**	**PAO1**			**Δ*pppA*-*ppk*A**		

chloramphenicol	25			55		
carbenicillin	35			75		

+ sign indicates positive reaction or manifestation

^a ^The pyoverdine values are expressed as OD_405 _values of culture supernatants normalized to cell density at OD_600_. Averages based on three independent assays are presented. The numbers in the parentheses represents standard errors of the means. The differences between the mutant and wild-type strain are statistically significant (*P *< 0.05).

^b ^The data are representative of four replicates.

### Oxidative and osmotic stress resistance of *P. aeruginosa *is affected by the Δ*pppA-ppkA *mutation

**Figure 2:**
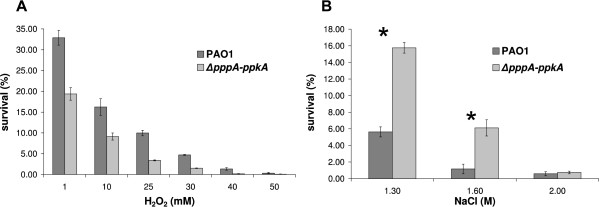
**Survival of *P. aeruginosa *PAO1 and Δ*ppp*A-*ppk*A strains upon exposure to environmental stresses**. **(A) Oxidative stress with 1 to 50 mM H_2_O_2_.** The differences between the mutant and its parental wild-type strain are statistically significant (*P *< 0.05) for all H_2_O_2 _concentrations. The standard errors of the means from three independent assays are shown. **(B) Hyperosmotic stress with 1.3 to 2 M NaCl**. The standard errors of the means from three independent assays are shown. The differences between the mutant and its parental wild-type strain are statistically significant (*P *< 0.05) for 1.3 and 1.6 M NaCl. An asterisk indicates a significant difference.

Resistance to oxidative stress is a highly important feature for *P. aeruginosa *during its infection of a lung affected by cystic fibrosis (CF). To characterise the ability of the *pppA-ppkA *mutant strain to cope with stress conditions, its ability to survive H_2_O_2_-induced oxidative stress was examined and compared with that of the parental strain. The sensitivity of stationary-phase cultures to oxidative stress was tested by exposing them to a range of H_2_O_2 _concentrations (1 mM to 50 mM) for 1 h. Decreased survival of the Δ*pppA-ppkA *mutant was observed compared to that of the wild-type beginning at 1 mM H_2_O_2 _exposure (Figure 2A). After 1 h of treatment with 1 mM hydrogen peroxide, only 20% of the Δ*pppA-ppkA *cells survived in comparison to 35% of the wild-type cells.

CF is characterised by disturbance in electrolyte transport which result in increased level of Na^+^, Cl^- ^and Ca^2+ ^[46]. Respiratory tract fluids in CF patients therefore presents environment with high osmolarity conditions. Therefore, we tested the ability of the Δ*pppA-ppkA *mutant strain to grow under high osmolarity conditions. Stationary-phase cells were treated with 1.3 to 2 M NaCl. The *pppA-ppkA *mutant cells showed increased resistance to an increased osmotic pressure caused by a high concentration of salt (Figure 2B). After 16 h of exposure, there was an approximately three-fold difference in relative viability between the parent and the mutant strain.

### The Δ*pppA-ppkA *mutant is more sensitive to macrophage-mediated killing than the wild-type strain

**Figure 3:**
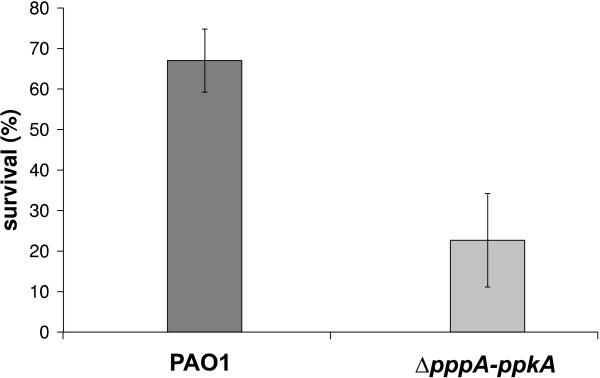
**Macrophage-mediated bactericidal assay**. Deletion of *pppA-ppkA *genes resulted in a significantly decreased survival rate. The standard errors of the means for 10 experimental points are shown. The differences between the mutant and its parental wild-type strain are statistically significant (*P *< 0.05). % of survival represents % of bacteria surviving after internalization.

Decreased resistance to oxidative stress, delayed production of several virulence factors, together with the decreased growth rate of the mutant led us to measure the sensitivity of bacterial cells to macrophage-mediated killing. Both the wild-type and mutant strains were incubated with murine J774 macrophages *in vitro*, and intracellular bacteria were recovered to determine the number of surviving bacterial cells. As shown in Figure 3, the Δ*pppA-ppkA *mutant is much more sensitive to macrophage-mediated killing than wild-type PAO1 cells, suggesting that the PpkA-PppA functional pair may regulate genes and/or proteins necessary for survival after exposure to macrophages. The mutant strain revealed a significantly decreased ability to survive (22.6%) in comparison to the wild-type strain (70%).

We suppose that highly increased sensitivity to oxidative killing is a major contributor to the survival of the mutant in a cellular bactericidal system because such cells will be less likely to survive the conditions inside the macrophage phagosomes.

### The Δ*pppA-ppkA *mutant strain is less susceptible to the antibiotics carbenicillin and chloramphenicol

*P. aeruginosa *is a major cause of nosocomial infections and is feared for its high intrinsic antibiotic resistance and its ability to develop multidrug resistance [47]. We analysed the sensitivity of the Δ*pppA-ppkA *mutant to different classes of antibiotics commonly used for the treatment of *P. aeruginosa *infections, including carbenicillin, pefloxacin, tetracycline, and trimethoprim, as well as chloramphenicol, which is used in resistance studies. The mutant strain demonstrated a greater than two-fold decrease in sensitivity to chloramphenicol and carbenicillin compared to the wild-type strain (Table 2). No significant changes were observed for the other antibiotics tested.

Taken together, the increased resistance of the mutant strain to osmotic stress and to the antibiotics chloramphenicol and carbenicillin suggests that PppA-PpkA pair might affect cellular functions connected with membrane permeability and transport.

### The mutation of *ppkA *and *pppA *compromises *P. aeruginosa *in the plant virulence model

The fact that the deletion of *pppA-ppkA *genes influenced stress tolerance and substantially decreased intracellular survival in the murine macrophages raised the possibility that it could also have significant implications in the ability of this mutant to cause disease. To assess the effect of the missing PppA-PpkA on the virulence of *P. aeruginosa*, we employed the lettuce leaf model of infection. Plants have previously been used as an *in vivo *pathogenesis model for the identification of *P. aeruginosa *virulence factors [37], and there are supporting data showing that the virulence mechanisms between plant and animal models are conserved [48].

**Figure 4:**
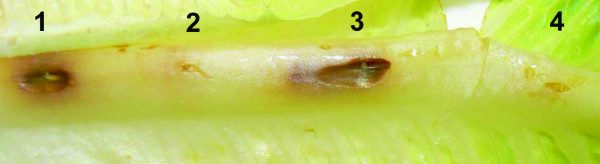
**Plant (lettuce) infection assay with the *P. aeruginosa *PAO1 (1, 3) and Δ*pppA*-*ppkA *(2, 4) strains**. The photograph shows a representative example of lettuce midribs after three days of infection. Infection by PAO1 shows necrosis and tissue maceration.

The pathogenicity assay revealed a significant difference in the manifestation of infection symptoms caused by the mutant compared to the wild-type strain. In contrast to the wild type, which induced severe necrotic lesions three days post-infection, the mutant strain did not cause any necrosis of the leaves even after a prolonged incubation period (Figure 4). This finding indicates that the protein kinase PpkA and its cognate phosphatase PppA regulate the cellular functions that are important for virulence in the plant model of infection.

### Transcriptional analysis of the *P. aeruginosa pppA-ppkA *mutant

To begin to address the genetic basis of these phenotypes, we performed a global transcription analysis of the Δ*pppA-ppkA *mutant compared to the wild-type parental strain in early stationary phase bacteria grown in M9 medium. *P. aeruginosa *whole-genome microarray GeneChips from Affymetrix (Materials and Methods) were used throughout this study. Among the 5,570 predicted open reading frames, changes in relative transcript levels with at least a 1.7-fold difference (with an adjusted *P*-value < 0.05) were observed for 83 genes. Of the 83 genes, 70 genes showed increased expression (by 1.7- to 12.7-fold) and 13 exhibited decreased expression (by 1.7- to 3.9-fold) in the Δ*pppA-ppkA *strain.

The affected genes were classified into three major groups constituting regulons: the oxidative stress-responsive genes [49-51], genes regulated by the stationary phase σ-factor RpoS [52] and/or by the *las *and *rhl *QS systems [53,54]; genes regulated by the Pseudomonas Quinolone Signal (PQS) [55,56] (Table 3).

**Table 3:** *P.aeruginosa *genes affected in the Δ*pppA-ppkA *mutant compared to the PAO1 wild-type strain

Gene (Name)	Fold change^a^	*P*-value	Protein (Function)^c^	Other regulators
***oxidative stress***				

***primary metabolism***				
PA0106 (*coxA*)^b^	2.58	0.177	cytochrome c oxidase, subunit I (Energy metabolism)	RpoS, QS
PA0107^b^	3.68	0.091	CHP (Energy metabolism)	RpoS, QS
PA0108 (*coIII*)	2.22	0.038	cytochrome c oxidase, transmembrabe helix, subunit III (Energy metabolism)	RpoS, QS
PA1317 (*cyoA*)	-3.08	0.013	cytochrome o ubiquinol oxidase subunit II (Energy metabolism)	QS
PA1318 (*cyoB*)	-2.79	0.009	cytochrome o ubiquinol oxidase subunit I (Energy metabolism)	QS
PA1319 (*cyoC*)	-2.39	0.043	cytochrome o ubiquinol oxidase subunit III (Energy metabolism)	QS
PA1320 (*cyoD*)	-2.27	0.041	cytochrome o ubiquinol oxidase subunit IV (Energy metabolism)	QS
PA1321 (*cyoE*)	-3.86	0.037	cytochrome o ubiquinol oxidase protein CyoE (Energy metabolism)	QS
***sulphur metabolism***				
PA0283 (*sbp*)^b^	4.89	0.061	sulfate-binding protein precursor, ABC transporter (Transport of small molecules)	
PA0284	10.61	0.038	HP	PQS
PA1493 (*cysP*)	4.73	0.004	sulfate-binding protein of ABC transporter (Transport of small molecules)	
PA1754 (*cysB*)	1.73	0.022	transcriptional regulator CysB (Amino acid biosynthesis and metabolism)	
PA1838 (*cysI*)^b^	2.80	0.051	sulfite reductase (Central intermediary metabolism)	
PA3441	2.76	0.027	Pr. molybdopterin-binding protein (Transport of small molecules)	
PA3450	8.46	0.047	Pr. antioxidant protein (Adaptation, protection) Probable Alkyl hydroperoxide reductase	
PA3935 (*tauD*)	1.81	0.037	taurine dioxygenase (Carbon compound catabolism)	RpoS
PA3936 (*tauC*)^b^	2.92	0.080	Pr. permease of ABC taurine transporter (Membrane proteins; Transport of small molecules)	RpoS
PA3938 (*tauA*)	5.07	0.025	Pr. periplasmic taurine-binding protein precursor (Transport of small molecules)	RpoS
PA4442 (*cysN*)^b^	3.13	0.117	ATP sulfurylase GTP-binding subunit/APS kinase (Amino acid biosynthesis and metabolism; Central intermediary metabolism)	QS
PA4443 (*cysD*)^b^	4.86	0.051	ATP sulfurylase small subunit (Amino acid biosynthesis and metabolism; Central intermediary metabolism)	QS
***others***				
PA0594 (*surA*)	2.03	0.036	peptidyl-prolyl cis-trans isomerase SurA (Adaptation, protection)	
PA1127^b^	-2.94	0.061	Pr. oxidoreductase (Adaptation, protection; Putative enzymes)	

***RpoS regulated genes***				

PA1708 (*popB*)^b^	-2.08	0.070	translocator protein PopB (Protein secretion/export apparatus)	QS, TTSS
PA1709 (*popD*)	-1.82	0.004	translocator protein PopD (Protein secretion/export apparatus)	QS, TTSS
PA4551 (*pilV*)	-2.09	0.008	type 4 fimbrial biogenesis protein PilV (Motility & Attachment)	
PA1048	2.80	0.037	Pr. outer membrane protein (Membrane proteins; Transport of small molecules)	
PA1119	1.95	0.021	Pr. outer membrane lipoprotein (Membrane proteins)	
PA1175 (*napD*)	1.88	0.021	NapD protein of periplasmic nitrate reductase (Energy metabolism)	QS
PA2204	1.87	0.014	Pr. binding protein component of ABC transporter (Transport of small molecules)	PQS
PA2223	2.76	0.021	HP	
PA2235	2.11	0.017	HP	
PA2303	1.80	0.035	HP	QS
PA2365	1.96	0.017	CHP	QS
PA2366	2.05	0.001	CHP	QS
PA2452	1.76	0.033	HP	
PA2570 (*pa1L*)	2.47	0.049	PA-I galactophilic lectin (Adaptation, protection; Motility & Attachment)	QS
PA2781	2.06	0.027	HP	
PA2939	4.49	0.018	Pr. aminopeptidase (Putative enzymes)	QS
PA3622 (*rpoS*)	3.86	0.030	sigma factor RpoS (Transcriptional regulators)	PQS
PA3848	1.93	0.020	HP	
PA4175 (*prpL*)	4.65	0.008	protease IV (Secreted Factors (toxins, enzymes, alginate))	QS
PA4296 (*pprB*)	3.69	0.029	PprB two-component response regulator (Transcriptional regulators; Two-component regulatory systems; Antibiotic resistance and susceptibility)	QS
PA4299 (*tadD*)^b^	2.41	0.086	tadD Flp pilus assembly protein (Function Motility & Attachment)	PprB, QS
PA4306 (*flp*)	6.69	0.002	Flp Type IVb pilin (Motility & Attachment)	PprB, QS

***QS regulated genes***				

PA0572	3.27	0.049	HP	
PA1874	2.02	0.048	HP	
PA2067	2.38	0.039	Pr. hydrolase (Putative enzymes)	
PA3724 (*lasB*)	2.23	0.037	elastase LasB (Translation, post-translational modification, degradation; Secreted Factors (toxins, enzymes, alginate))	RpoS, PQS
PA3922	11.31	0.038	CHP	
PA3923^b^	8.11	0.143	HP	
PA4497	2.61	0.031	Pr. binding protein component of ABC transporter (Transport of small molecules)	
PA5481	2.01	0.038	HP	

***PQS/MvfR regulon***				

PA0200	5.10	0.048	HP	
PA0201	5.10	0.048	HP	
PA0996 (*pqsA*)	4.56	0.011	Pr. coenzyme A ligase (Biosynthesis of cofactors, prosthetic groups and carriers)	
PA0997 (*pqsB*)	2.05	0.007	Homologous to beta-keto-acyl-acyl-carrier protein synthase (Biosynthesis of cofactors, prosthetic groups and carriers)	
PA0998 (*pqsC*)	2.07	0.038	Homologous to beta-keto-acyl-acyl-carrier protein synthase (Biosynthesis of cofactors, prosthetic groups and carriers)	
PA0999 (*pqsD*)	2.24	0.009	3-oxoacyl-[acyl-carrier-protein] synthase III (Biosynthesis of cofactors, prosthetic groups and carriers)	
PA1000 (*pqsE*)	2.50	0.016	Quinolone signal response protein (Biosynthesis of cofactors, prosthetic groups and carriers)	
PA1001 (*phnA*)^b^	2.54	0.065	anthranilate synthase component I (Adaptation, protection)	
PA1002 (*phnB*)^b^	2.19	0.071	anthranilate synthase component II (Adaptation, protection; Amino acid biosynthesis and metabolism	
PA3520	2.31	0.020	HP	oxidative stress
PA4377	3.01	0.022	HP	

***other genes***				

PA4139	12.71	0.018	HP	

^a ^Fold changes represent the ratio of the expression levels in the comparison of *P. aeruginosa *Δ*pppA-ppkA *and wild type PAO1. Minus (-) sign indicates a decreased expression in Δ*pppA-ppkA *mutant strain;^b ^*p*-value > 0.05, only genes from the same transcriptional unit are listed;^c ^HP - hypothetical protein, CHP- conserved hypothetical protein, Pr. - probable

The microarray results were validated by the RT-PCR analysis. PA2570 (*pa1L*), PA4296 (*pprB*), PA4175 (*prpL*), PA3724 (*lasB*), PA3622 (*rpoS*), PA0996 (*pqsA*), PA1001 (*phnA*), PA1317 (*cyoA*), PA1754 (*cysB*), PA1127, and PA4139 transcripts were quantified as representatives of the detected regulons and a range of mRNA level changes. In each instance, the RT-PCR results correlated well (R^2 ^= 0.9) with those obtained from the microarrays (Table 4).

**Table 4:** Transcript level comparison of *P.aeruginosa *genes between microarray analysis and real-time PCR analysis

non-stress conditions			oxidative stress conditions		
	**mRNA level change^a^**			**mRNA level change^a^**	
**Gene (name)**	**microarray**	**real-time PCR**	**Gene (name)**	**microarray**	**real-time PCR**

PA2570 (*pa1L*)	2.47	2.71 (± 0.45)	PA4764 (*fur*)	-2.44	-1.66 (± 0.04)
PA4296 (*pprB*)	3.69	3.71 (± 0.39)	PA4227 (*pchR*)	2.47	1.60 (± 0.23)
PA4175 (*prpL*)	4.65	3.63 (± 0.38)	PA2384	3.11	2.01 (± 0.15)
PA3724 (*lasB*)	2.23	2.98 (± 0.27)	PA1317 (*cyoA*)	3.12	3.00 (± 0.27)
PA3622 (*rpoS*)	3.86	4.4 (± 0.45)	PA4471 (*fagA*)	13.62	7.51 (± 0.61)
PA0996 (*pqsA*)	4.56	2.74 (± 0.12)	PA3617 (*recA*)	-2.26	-1.54 (± 0.04)
PA1001 (*phnA*)	2.54	1.51 (± 0.19)	PA3007 (*lexA*)	-2.02	-1.98 (± 0.02)
PA1317 (*cyoA*)	-3.08	-2.59 (± 0.08)	PA5360 (*phoB*)	-2.00	-1.82 (± 0.06)
PA1754 *(cysB*)	1.73	2.75 (± 0.19)	PA2165	3.96	3.45 (± 0.4)
PA1127	-2.94	-5.00 (± 0.04)	PA0073 (*tagT1*)	44.76	29.55 (± 3.92)
PA4139	12.71	13.3 (± 0.71)	PA0072 (*tagS1*)	5.15	3.67 (± 0.7)

^a ^Fold changes represent the ratio of the expression levels in the comparison of *P. aeruginosa *Δ*pppA-ppkA *and wild type PAO1. Minus (-) sign indicates a decreased expression in Δ*pppA-ppkA *mutant strain.

#### Oxidative stress-responsive genes

The genes encoding proteins involved in oxidative stress response comprised the largest category, containing genes with the most considerable alterations in relative transcription levels. These genes were identified during experiments that tested *P. aeruginosa*'s response to hydrogen peroxide [49-51]. In addition, many of these genes were also found to have altered expression levels in a stationary phase σ-factor RpoS mutant [52], were found to be regulated by QS [53,54], or were genes that are affected by the addition of PQS to *P. aeruginosa *cultures [55].

##### Oxidative stress-responsive genes of primary metabolism

Genes of primary metabolism were previously shown to be important in combating oxidative stress through the production of cofactors such as NADPH, an essential cofactor for glutathione reductases, and alkyl hydroperoxide reductases, genes of the adaptive response to oxidative stress [57].

Repressed genes were represented by the *cyoABCDE *operon, which encodes cytochrome o ubiquinol oxidase, the main terminal oxidase of the electron transport chain under highly aerobic conditions [58]. On the contrary, cytochrome c oxidase complex genes (*coxA*, PA0107, *coIII*), which form the main aerobic respiration system, showed increased expression. As was shown by Salunkhe et al., the *cyoABCD *operon is highly upregulated during oxidative stress, whereas *cox *genes are repressed [51]. In our study, the regulation of these two operons was reversed, which could imply that the mutant strain is not able to cope optimally with oxidative stress. This fact could contribute to the decreased resistance of the mutant strain to oxidative stress.

##### Oxidative stress-responsive genes of sulphur metabolism

Another important element affected by oxidative stress is sulphur, an essential part of iron-sulphur proteins which are particularly prone to oxidative damage [59]. The expression of several genes involved in sulphur metabolism is upregulated in the mutant strain, including sulphate binding proteins Sbp and CysP, sulphite reductase CysI, and the ATP sulphurylase, CysND. In addition, the hypothetical protein gene PA0284, which is upstream of *sbp*, was one of the most upregulated genes (more than 10-fold) in our study. We also observed increased expression of the *cysB *and CysB-regulated genes. CysB transcriptional regulator is required for growth with a variety of organosulphur compounds and is known to regulate sulphur metabolism and transport genes [60,61] (see Table 3).

#### Genes of the RpoS regulon

Our study revealed increased expression of the *rpoS *gene (four-fold increase), which codes for stationary phase σ-factor RpoS, and alterations in expression of genes dependent on RpoS [52]. Many of the genes regulated by RpoS have been also reported as simultaneously affected by the *las/rhl *QS systems [53,54].

Among the genes of this regulon with decreased expression were those encoding the components of the type III secretion system, including PA1708-9 (*popBD*), as well as a gene involved in the production of type IV pili, PA4551 (*pilV*), which is coregulated by TTSS [28]. We observed also approximately 2-fold decrease in expression of PA3841 (*exoS*) and PA0044 (*exoT*) genes, however, their *P *values were above 0.1 level (0.188 and 0.113, respectively). These findings corresponded to the results of Hogardt et al. [62], who observed increased expression of *exoS *in both *rhl *and *rpoS *mutants.

The expression of the virulence factor genes PA2570 (galactophilic lectin PA-I) and PA4175 (*prpL*, protease IV) was increased. The remaining genes of the RpoS regulon were represented mainly by genes coding for hypothetical proteins with unknown function.

Upregulated gene PA4296, which codes for the two-component response regulator PprB, was shown to be regulated by both RpoS [52] and QS [53]. Consequently, the increased expression of PprB resulted in an increased expression of PA4299 (*tadD*) and PA4306 (*flp*). PprB was shown to positively regulate the expression of the *tad *locus, which is responsible for type IVb pili assembly [63]. Type IVb pili are required for adhesion to abiotic surfaces and to eukaryotic cells [63].

#### QS and PQS regulated genes

Another functional category of affected genes is comprised of those whose expression is mediated by the QS mechanism. However, no alterations in the expression levels of key QS regulator genes, including *lasI*, *lasR*, *rhlI *and *rhlR*, were observed. Therefore, it is likely that QS-dependent genes might be indirectly regulated by PppA-PpkA through RpoS and/or PQS, which were differentially expressed in the mutant strain. Interestingly, gene coding for elastase LasB was upregulated 2.2-fold. However, no difference in elastolytic activity was found by Elastin-Congo Red assay. In addition, a notably large increase in the expression of the genes PA3922-3923, which encode hypothetical proteins, was detected.

The genes encoding proteins involved in PQS biosynthesis comprised large category, containing seven genes that were coordinately upregulated 2.2- to 4.6-fold. These genes are organised in two putative transcriptional units, *pqsABCDE *and *phnAB*, and are regulated by the PQS synthesis regulator, MvfR [56].

Other affected genes were classified into a wide variety of functional categories, including genes involved in primary metabolism, the transport of small molecules, genes encoding ribosomal proteins and hypothetical genes. The most upregulated gene in our study was gene PA4139 with unknown function. It was shown by Aespedon et al. [64] as upregulated under osmotic stress conditions. Therefore, it is likely that its overexpression is related to increased resistance of the *ΔpppA-ppkA *to osmotic stress. This gene is highly upregulated also under conditions of oxidative stress (13.54).

### Global transcriptome analysis under oxidative stress conditions

The results of the transcriptomic profile analysis showed that a considerable number of genes involved in the oxidative stress response were differentially expressed in the mutant strain when compared to the wild-type strain. In addition, the genes from this group were the most affected genes in our study. Because phenotypic studies showed that the mutant strain had increased sensitivity to oxidative stress, these results prompted us to analyse the transcription profiles of both strains under conditions of oxidative stress.

The wild-type and mutant strains were grown under the same conditions as the first analysis up to OD_600 _= 0.8, at which time sublethal oxidative stress was generated by the addition of hydrogen peroxide. Sublethal conditions were proven by measuring bacterial survival 15 min after the addition of 10 mM H_2_O_2_, which was determined by plating bacterial cultures on LB plates. No significant differences in survival rate were observed between initial and end points in either the mutant or parent strain (data not shown).

Analysis of the transcriptomes of the PAO1 and Δ*pppA-ppkA *mutant strain revealed 261 genes that were significantly differentially regulated by at least two-fold with an adjusted *P *value ≤ 0.05 for *t*-test. Only spots that were present in at least three out of four samples were used for further analysis. Of the 261 genes, 131 genes showed increased expression (by 2- to 44.7-fold) and 130 showed decreased expression (by 2- to 7.7-fold) in the Δ*pppA-ppkA *strain.

To validate the transcriptomic data, we selected representatives of affected genes, including PA4764 (*fur*), PA4227 (*pchR*), PA2384, PA1317 (*cyoA*), PA4471 (*fagA*), PA3617 (*recA*), PA3007 (*lexA*), PA5360 (*phoB*), PA2165, PA0073, and PA0072, covering a range of mRNA level changes and determined their expression by independent quantitative RT PCR analysis. As shown in Table 4, the data obtained by the Affymetrix Pseudomonas GeneChip array analysis were again corroborated with RT-PCR analyses of the selected genes (R^2 ^= 0.97).

#### Genes related to the response to oxidative stress

As expected, a majority of affected genes were related to the oxidative stress response. A similar expression pattern has been described in previous studies of the oxidative stress response [49-51]. These genes can be classified further as genes of the adaptive stress response (e.g., genes of the SOS regulon, genes related to protective cellular mechanisms, pyocin genes), genes related to iron regulation, and primary metabolism genes.

##### SOS regulon

DNA damage caused by hydroxyl radicals induces the expression of the SOS regulon repressor *lexA *gene and *recA*. RecA stimulates the cleavage of LexA to allow the expression of SOS regulon genes such as the inhibitors of cell division PA3008 and PA0671 or damage inducible protein P gene PA0923 (*dinB*). All genes were significantly less upregulated in the mutant strain than in the wild type.

##### The genes related to protective cellular mechanisms

The genes related to protective cellular mechanisms were represented by alkyl hydroperoxide reductase genes *ahpC*, *ahpF *and PA0848. The expression levels of all of these genes were considerably less induced in the mutant than in the wild-type strain. For instance, the relative transcript level of the alkyl hydroperoxide reductase PA0848 gene was 5.2-fold increased in the mutant strain whereas expression was increased 29-fold in the wild type. Expression of other genes involved in the adaptation to oxidative stress encoded by the *fagA*-*fumC*-*orfX*-*sodM *operon (PA4468-71) was also affected. The expression of this operon was shown to be repressed by ferric uptake regulation protein (Fur) and induced by iron starvation [65]. In our study, expression of the genes from this operon, as well as other Fur repressed genes, was significantly less downregulated in the mutant strain when compared to the wild type (discussed below). Our findings suggest that the response of the mutant strain to the oxidative stress is deregulated, resulting in more extensive cellular damage caused by reactive hydroxyl radicals as demonstrated by phenotypic studies.

##### Pyocin genes

Hypothesis of increased cellular damage is further strengthened by uniformly higher transcript levels of pyocin genes (PA0614-PA0646) in the mutant strain when compared to the PAO1 parental strain. F-, R- and S- type pyocins, bacteriocins of *P. aeruginosa*, were shown to be strongly induced in response to hydrogen peroxide [49,66]. Pyocins cause cell death through DNA breakdown and the inhibition of lipid synthesis. Pyocin S5 has a pore-forming activity. Their production is inducible by treatments that cause DNA damage and the subsequent activation of RecA, which co-regulates the activity of many pyocin genes [67].

##### The oxidative stress response is intimately linked with iron homeostasis

As shown by Chang et al. [49], the expression of genes regulated by Fe^2+ ^metabolism is repressed during oxidative stress by the ferric uptake regulator, Fur, to prevent further generation of hydroxyl radicals from H_2_O_2_. Consistently, expression of *fur *gene in the wild-type strain was increased, whereas the mutant strain showed decreased expression (*P *value 0.086; verified by RT-PCR, see Table 4 and 5). As a consequence, genes of siderophore pyochelin biosynthesis, *pchDCB*, and regulator *pchR *were less downregulated. The genes involved in the synthesis of siderophore pyoverdine [68] showed divergent expression which could be result of less downregulated gene PA2384 encoding global regulator responsive to Fe^2+ ^[69]. The genes PA2381, PA2384, and PA2398 were less downregulated in the mutant strain, whereas genes PA2403-2404 and PA2409 which were not identified as PA2384-dependent [69] were more downregulated in the mutant strain. The mRNA levels of other iron-regulated genes such as *tonB*, which codes for ferrisiderophore receptor protein TonB, *fpvA *gene, which encodes ferripyoverdine receptor, and the probable TonB-dependent receptor gene PA5505 were less decreased.

**Table 5:** *P.aeruginosa *genes affected in the Δ*pppA-ppkA *mutant compared to PAO1 wild-type strain under the conditions of oxidative stress

					Normalized gene signals^d^			
Gene (name)	Fold change^b^	*P*-value	Protein (function)^c^	Other regulators	Δ H_2_O_2_	Δ	wt H_2_O_2_	wt
***Oxidative stress-responsive genes***								

***Genes of SOS regulon***								
PA0669	-2.85	0.027	Pr. DNA polymerase alpha chain (DNA replication, recombination, modification and repair; Putative enzymes)		4.4	3.5	5.9	3.7
PA0671	-3.58	0.000	HP		6.1	2.4	8.0	2.4
PA0923 (*dinB*)	-2.42	0.037	DNA damage inducible protein P (DNA replication, recombination, modification and repair; Adaptation, protection)		5.7	3.4	7.0	3.3
PA2288	-2.24	0.003	HP		8.7	3.2	9.9	3.7
PA3007 (*lexA*)	-2.02	0.023	repressor protein LexA (Adaptation, protection; Translation, post-translational modification, degradation)		8.0	4.8	9.1	5.0
PA3008	-2.26	0.010	HP		7.2	3.1	8.4	2.8
PA3617 (*recA*)	-2.26	0.020	RecA protein (DNA replication, recombination, modification and repair)		10.1	7.6	11.2	7.5
PA0911	2.81	0.010	HP		4.4	2.7	2.9	2.8
***Genes of protective cellular mechanisms***								
PA0139 (*ahpC*)^a^	-1.91	0.090	alkyl hydroperoxide reductase subunit C (Adaptation, protection)		11.9	11.3	12.8	10.2
PA0140 (*ahpF*)	-2.73	0.044	alkyl hydroperoxide reductase subunit F (Adaptation, protection)		6.7	3.8	8.1	3.6
PA0848	-7.13	0.001	Pr. alkyl hydroperoxide reductase (Adaptation, protection; Putative enzymes)		4.9	2.5	7.7	2.8
PA2825	-3.08	0.001	Pr. transcriptional regulator (Two-component regulatory systems)		6.8	3.7	8.4	3.7
PA2826	-2.32	0.018	Pr. glutathione peroxidase (Adaptation, protection)		8.2	5.4	9.4	5.3
PA2868	-7.76	0.001	HP (Membrane proteins)		4.4	2.8	7.3	2.9
PA4400	-2.60	0.012	Pr. pyrophosphohydrolase (DNA replication, recombination, modification and repair)		3.2	3.0	4.5	2.9
PA4612 (*ankB*)	-2.79	0.042	CHP		3.7	2.4	5.2	2.5
PA1127	-3.07	0.032	Pr. oxidoreductase (Adaptation, protection; Putative enzymes)		6.0	6.5	7.6	8.3
PA3287	-6.70	0.001	CHP	RpoS	6.6	3.9	9.4	4.0
PA4468 (*sodM*)	3.36	0.015	superoxide dismutase (Adaptation, protection)		8.9	10.8	7.2	10.4
PA4469 (*orfX*)	9.73	0.001	HP		9.4	11.8	6.1	11.1
PA4470 (*fumC1*)	7.86	0.002	fumarate hydratase (Energy metabolism)		8.8	11.3	5.8	10.5
PA4471 (*fagA*)	13.62	0.005	fagA/HP		9.5	10.7	5.8	10.3
***Genes of pyocin synthesis***								
PA0614	3.60	0.009	HP		6.6	3.9	4.7	4.0
PA0617	4.79	0.002	Pr. bacteriophage protein		7.4	4.6	5.2	4.9
PA0618	3.05	0.027	Pr. bacteriophage protein (Related to phage, transposon, or plasmid)		7.1	5.0	5.5	5.1
PA0619	3.53	0.039	Pr. bacteriophage protein (Related to phage, transposon, or plasmid)		7.8	5.3	5.9	4.9
PA0620	2.51	0.035	Pr. bacteriophage protein (Related to phage, transposon, or plasmid)		6.7	4.9	5.4	4.8
PA0622	2.56	0.029	Pr. bacteriophage protein (Related to phage, transposon, or plasmid)		7.0	5.8	5.7	5.2
PA0623	3.44	0.042	Pr. bacteriophage protein (Related to phage, transposon, or plasmid)		8.4	6.5	6.6	5.9
PA0624	3.01	0.014	HP (Related to phage, transposon, or plasmid)		6.9	4.7	5.3	4.4
PA0625	3.02	0.009	HP (Related to phage, transposon, or plasmid)		5.5	4.2	3.9	4.0
PA0626^a^	2.35	0.053	HP (Related to phage, transposon, or plasmid)		5.5	3.8	4.3	3.5
PA0627	3.03	0.006	CHP (Related to phage, transposon, or plasmid)		5.6	3.9	4.0	3.9
PA0628	3.97	0.004	CHP (Related to phage, transposon, or plasmid)		6.8	4.8	4.8	4.0
PA0629	3.62	0.046	CHP (Related to phage, transposon, or plasmid)		5.7	3.1	3.9	3.1
PA0630	3.88	0.034	HP (Related to phage, transposon, or plasmid)		6.8	4.7	4.8	4.0
PA0631	4.01	0.032	HP (Related to phage, transposon, or plasmid)		6.0	3.5	4.0	3.1
PA0632	2.95	0.001	HP (Related to phage, transposon, or plasmid)		4.0	2.4	2.5	2.4
PA0635	3.69	0.034	HP (Related to phage, transposon, or plasmid)		6.3	3.5	4.4	3.5
PA0636	4.46	0.004	HP (Related to phage, transposon, or plasmid)		7.4	5.3	5.2	4.8
PA0637	2.97	0.006	CHP (Related to phage, transposon, or plasmid)		5.1	3.1	3.5	2.9
PA0638	4.28	0.003	Pr. bacteriophage protein (Related to phage, transposon, or plasmid)		6.0	3.6	3.9	3.3
PA0639	4.28	0.022	CHP (Related to phage, transposon, or plasmid)		6.3	3.7	4.2	3.4
PA0640	3.42	0.003	Pr. bacteriophage protein (Related to phage, transposon, or plasmid)		4.3	2.3	2.6	2.3
PA0641	3.02	0.008	Pr. bacteriophage protein (Related to phage, transposon, or plasmid)		5.2	3.2	3.6	3.1
PA0643	2.07	0.011	HP (Related to phage, transposon, or plasmid)		3.7	2.5	2.7	2.4
PA0646	2.26	0.049	HP (Related to phage, transposon, or plasmid)		4.4	3.2	3.3	3.0
PA3142	-2.62	0.015	HP (Related to phage, transposon, or plasmid)		6.7	5.9	8.1	5.5
PA3143	-2.92	0.002	HP (Related to phage, transposon, or plasmid)		3.4	3.0	5.0	2.9
***Iron metabolism related genes***								
Pyoverdine								
PA2381	4.72	0.023	HP		6.9	8.4	4.7	8.7
PA2384	3.11	0.006	transcriptional regulator		7.8	10.8	6.2	10.5
PA2403	-2.96	0.032	HP (Membrane proteins)		3.7	5.9	5.2	5.8
PA2404	-2.19	0.019	HP (Membrane proteins)		6.3	8.2	7.4	7.9
PA2405	-2.14	0.023	HP		5.0	7.5	6.1	7.6
PA2409	-2.58	0.015	Pr. permease of ABC transporter (Membrane proteins; Transport of small molecules)		4.1	6.6	5.4	6.1
Pyochelin								
PA4227 (*pchR*)^a^	2.47	0.098	transcriptional regulator PchR (Transcriptional regulators)		7.0	7.2	5.7	7.4
PA4228 (*pchD*)	6.12	0.029	pyochelin biosynthesis protein PchD (Transport of small molecules; Secreted Factors (toxins, enzymes, alginate))		8.4	10.9	5.7	9.8
PA4229 (*pchC*)	5.89	0.059	pyochelin biosynthetic protein PchC (Transport of small molecules; Secreted Factors (toxins, enzymes, alginate))		9.1	11.4	6.6	10.5
PA4230 (*pchB*)	6.08	0.038	salicylate biosynthesis protein PchB (Transport of small molecules; Secreted Factors (toxins, enzymes, alginate))		8.3	11.9	5.7	11.1
Others								
PA1300	3.86	0.028	Pr. sigma-70 factor, ECF subfamily (Transcriptional regulators)		6.4	7.0	4.5	7.3
PA4211	2.74	0.007	Pr. phenazine biosynthesis protein (Secreted Factors (toxins, enzymes, alginate))		4.1	10.8	2.6	10.0
PA4570	5.46	0.011	HP Pr. negative regulator		9.2	11.9	6.7	11.8
PA5505	2.27	0.017	Pr. TonB-dependent receptor (Membrane proteins; Transport of small molecules)		3.8	7.0	2.7	5.8
PA5531 (*tonB*)	2.25	0.043	TonB protein (Transport of small molecules)		9.2	9.6	8.1	9.6
PA4764 (*fur*)^a^	-2.44	0.086	ferric uptake regulation protein (Transcriptional regulators)		6.0	7.4	7.3	6.3
PA4895	-2.35	0.039	Pr. transmembrane sensor (Transcriptional regulators; Membrane proteins)		3.2	3.9	4.4	4.4
***Primary metabolism***								
PA0105 (*coxB*)	-2.38	0.048	cytochrome c oxidase, subunit II (Energy metabolism)		8.9	5.7	10.1	4.9
PA0108 (*coIII*)^a^	-1.89	0.062	cytochrome c oxidase, subunit III (Energy metabolism)		9.4	6.0	10.3	4.6
PA0250	-4.17	0.001	CHP		5.7	5.4	7.8	5.6
PA1756 (*cysH*)	-2.16	0.007	3'-phosphoadenosine-5'-phosphosulfate reductase (Amino acid biosynthesis and metabolism)		4.0	5.3	5.1	4.5
PA2611 (*cysG*)	-2.02	0.008	siroheme synthase (Biosynthesis of cofactors, prosthetic groups and carriers)		4.9	4.7	5.9	4.8
PA2869	-3.36	0.000	HP		2.6	2.6	4.4	2.7
PA0603^a^	2.50	0.095	Pr. ATP-binding component of ABC transporter (Transport of small molecules)		7.9	4.8	6.5	4.1
PA0604	3.46	0.012	Pr. binding protein component of ABC transporter (Transport of small molecules)		5.0	2.7	3.2	2.5
PA0605	2.83	0.003	Pr. permease of ABC transporter (Membrane proteins); Transport of small molecules)		5.6	3.0	4.1	3.0
PA1317 (*cyoA*)	3.12	0.026	cytochrome o ubiquinol oxidase subunit II (Energy metabolism)		7.1	8.3	5.5	9.7
PA1318 (*cyoB*)	2.24	0.032	cytochrome o ubiquinol oxidase subunit I (Energy metabolism)		4.4	5.8	3.2	7.3
PA1319 (*cyoC*)	3.10	0.026	cytochrome o ubiquinol oxidase subunit III (Energy metabolism)		5.2	6.1	3.6	7.4
PA1321 (*cyoE*)	2.21	0.040	cytochrome o ubiquinol oxidase protein CyoE (Energy metabolism)		5.7	5.7	4.6	7.5
PA2646 (*nuoK*)	2.24	0.043	NADH dehydrogenase I chain K (Energy metabolism)		4.9	5.4	3.7	5.5
PA3441	7.35	0.006	Pr. molybdopterin-binding protein (Transport of small molecules)		9.5	7.2	6.6	5.6
PA3442^a^	2.69	0.092	Pr. ATP-binding component of ABC transporter (Transport of small molecules)		7.5	6.4	6.0	5.1
PA5170 (*arcD*)	4.45	0.049	arginine/ornithine antiporter (Amino acid biosynthesis and metabolism; Membrane proteins; Transport of small molecules)		8.1	7.4	6.0	6.7
PA5171 (*arcA*)	2.67	0.019	arginine deiminase (Amino acid biosynthesis and metabolism)		7.6	9.0	6.2	8.5
PA5172 (*arcB*)	2.66	0.026	ornithine carbamoyltransferase, catabolic (Amino acid biosynthesis and metabolism)		7.3	8.8	5.9	8.5
***Others***								
PA3237	-2.89	0.004	HP		3.9	2.4	5.5	2.5
PA0157 (*triB*)	2.28	0.001	Pr. RND efflux membrane fusion protein precursor (Transport of small molecules)		6.6	5.3	5.4	5.4

***QS regulated genes***								

***PA2134-92 locus***								
PA2134	4.16	0.032	HP		8.7	5.6	6.6	5.7
PA2135	2.85	0.027	Pr. transporter (Membrane proteins; Transport of small molecules)		4.7	3.0	3.2	3.4
PA2136	3.01	0.004	HP		4.1	2.3	2.5	2.4
PA2139	6.55	0.001	HP		6.8	2.6	4.1	2.8
PA2140	3.74	0.018	Pr. metallothionein (Central intermediary metabolism)		6.8	3.0	4.9	3.4
PA2141	5.64	0.003	HP		6.9	2.8	4.4	2.8
PA2142	4.05	0.029	Pr. short-chain dehydrogenase (Putative enzymes)		7.7	3.9	5.7	4.3
PA2144 (*glgP*)	4.01	0.008	glycogen phosphorylase (Cell wall/LPS/capsule)		6.8	3.7	4.8	3.6
PA2148	4.37	0.016	CHP (Membrane proteins)		8.1	4.2	6.0	4.3
PA2149	6.01	0.011	HP		9.4	4.6	6.9	4.7
PA2150	5.76	0.000	CHP		5.5	2.4	2.9	2.4
PA2151	3.46	0.041	CHP		7.0	3.6	5.2	3.4
PA2156	2.40	0.001	CHP		3.6	2.4	2.4	2.4
PA2157	4.03	0.002	HP		6.2	3.4	4.2	3.8
PA2158	4.51	0.019	Pr. alcohol dehydrogenase (Zn-dependent) (Putative enzymes)		7.9	4.1	5.7	4.0
PA2159^a^	2.89	0.062	CHP		5.9	3.2	4.4	3.7
PA2161^a^	3.33	0.081	HP		5.1	4.3	3.4	4.2
PA2162	2.14	0.049	Pr. glycosyl hydrolase (Putative enzymes)		6.2	4.6	5.1	4.8
PA2163	2.79	0.023	HP		8.3	5.4	6.8	5.3
PA2164	4.66	0.026	Pr. glycosyl hydrolase (Putative enzymes)		9.6	5.7	7.4	5.1
PA2165	3.96	0.008	Pr. glycogen synthase (Energy metabolism)		10.3	5.5	8.3	5.3
PA2167	2.72	0.049	HP		6.5	3.0	5.1	2.5
PA2168	2.78	0.049	HP		8.0	4.6	6.5	4.3
PA2178	3.55	0.004	HP		6.6	3.3	4.8	3.9
PA2179	3.16	0.023	HP		5.7	2.5	4.1	2.7
PA2180	3.05	0.032	HP		8.3	4.2	6.6	4.2
PA2181	2.99	0.001	HP		5.3	2.6	3.7	2.8
PA2184	5.62	0.007	CHP		7.6	4.9	5.1	5.0
PA2187	3.01	0.038	HP		7.2	3.4	5.6	3.9
PA2188	2.14	0.032	Pr. alcohol dehydrogenase (Zn-dependent) (Putative enzymes)		4.2	2.8	3.1	2.8
PA2192	2.72	0.020	CHP		5.4	2.9	4.0	3.0
***QS regulated virulence factors***								
PA1246 (*aprD*)^a^	2.10	0.066	alkaline protease secretion protein AprD (Secreted Factors (toxins, enzymes, alginate); Protein secretion/export apparatus)		6.4	8.0	5.4	8.1
PA1248 (*aprF*)^a^	2.05	0.072	alkaline protease secretion protein AprF (Protein secretion/export apparatus; Secreted Factors (toxins, enzymes, alginate))		6.4	6.8	5.4	6.6
PA1249 (*aprA*)	3.25	0.042	alkaline metalloproteinase precursor (Secreted Factors (toxins, enzymes, alginate)	PhoB	6.6	8.3	4.9	7.2
PA2300 (*chiC*)	6.31	0.004	chitinase (Carbon compound catabolism)	RpoS	5.7	8.3	3.1	7.2
PA2570 (*pa1L*)	3.37	0.025	PA-I galactophilic lectin (Adaptation, protection; Motility & Attachment)	RpoS	6.6	5.8	4.8	4.8
PA3477 (*rhlR*)	2.25	0.013	transcriptional regulator RhlR (Adaptation, protection; Transcriptional regulators)	RpoS	8.9	9.2	7.8	9.3
PA3724 (*lasB*)	2.37	0.042	elastase LasB (Translation, post-translational modification, degradation; Secreted Factors (toxins, enzymes, alginate))	RpoS PQS	6.0	8.4	4.7	6.6

***PhoB regulon***								

PA2364	-2.65	0.006	HP	RpoS	5.6	5.4	7.0	5.2
PA2560	-2.09	0.039	HP		5.1	3.8	6.2	3.0
PA2931 (*cifR*)	-2.05	0.008	transcriptional regulator (Transcriptional regulators)		5.4	3.6	6.5	4.2
PA3006 (*psrA*)	-2.31	0.049	transcriptional regulator PsrA (Transcriptional regulators)		5.1	4.4	6.3	5.1
PA5360 (*phoB*)	-2.00	0.004	two-component response regulator PhoB (Transcriptional regulators; Two-component regulatory systems)		4.6	3.5	5.6	3.5
PA5365 (*phoU*)	-1.76	0.012	phosphate uptake regulatory protein PhoU (Membrane proteins; Transcriptional regulators)		6.1	4.1	6.9	4.1
PA5369 (*pstS*)	-2.03	0.049	phosphate ABC transporter, periplasmic phosphate-binding protein (Transport of small molecules)		8.3	5.2	9.3	5.3
PA3309	3.88	0.010	CHP		7.7	7.3	5.7	7.2

***Other genes***								

PA3757	-5.59	0.005	Pr. transcriptional regulator (Transcriptional regulators)		3.4	3.4	5.9	2.8
PA2414	2.70	0.039	L-sorbosone dehydrogenase (Carbon compound catabolism)		6.2	5.2	4.8	4.5
PA2416 (*treA*)	3.31	0.025	periplasmic trehalase precursor (Carbon compound catabolism)		6.5	3.7	4.8	4.0
PA4139	13.54	0.044	HP		7.2	8.0	3.5	4.2

^a ^*p*-value > 0.05, only genes from the same transcriptional unit are listed;^b ^Fold changes represent the ratio of the expression levels in the comparison of *P. aeruginosa *Δ*pppA-ppkA *and wild type PAO1 under the conditions of oxidative stress. Minus (-) sign indicates a decreased expression in Δ*pppA-ppkA *mutant strain;^c ^HP - hypothetical protein, CHP- conserved hypothetical protein, Pr. - probable^ d ^ Means of normalized gene signals of two replicates; Δ H_2_O_2_/Δ - mutant strain signal intensities treated/untreated with 10 mM H_2_O_2_; wt H_2_O_2_/wt - wild-type PAO1 strain signal intensities treated/untreated with 10 mM H_2_O_2_

##### Oxidative stress affects the expression of primary metabolism genes

Our comparison of mRNA levels of the cytochrome c oxidase genes *coxB *and *coIII*, components of the terminal energy-transducing respiratory complex, showed that the increase of their expression was less profound in the mutant than in the wild-type strain. A similar trend was found in the case of the *cyoABCDE *operon, which codes for the main terminal oxidase of the electron transport chain under high oxygen tension [70]. Similarly, the expression of other energy metabolism genes was less decreased, as was case for *nuoK*, which is part of complex I of the respiratory chain, or *arcDAB *of arginine fermentation, which is one of the major pathways of anaerobic energy metabolism in *P. aeruginosa*.

In conclusion, analysis of the transcript levels of genes related to the oxidative stress response showed that the response of the mutant strain Δ*pppA-ppkA *is generally less proficient than the wild-type strain, which might result in the increased damage evidenced by the increased expression of pyocin genes and significantly reduced survival of the mutant strain when grown in the presence of a wide range of H_2_O_2 _concentrations.

#### QS/RpoS regulated genes

A number of genes identified in our study were previously found to be regulated by a quorum sensing mechanism [53]. Transcript levels of almost all of these genes were higher in the mutant strain than in the wild type. The majority of affected genes fall into the large cluster spanning the genes from PA2134 to PA2192, which were found to be regulated by the *rhl *quorum sensing system [53] and upregulated by exposure to human respiratory epithelia [71] in the PAO1 strain. Under oxidative stress, all 31 genes in this cluster were upregulated 2.1- to 6.5-fold. However, very little is known about the function of genes encoded by this cluster; 22 out of 31 affected genes are hypothetical, unclassified, and unknown. The computationally predicted operons PA2165-PA2160 and PA2151-PA2153 contain several genes related to synthesis of trehalose and glycogen. Trehalose accumulates in many bacteria as a compatible solute at high osmolarity and plays a role in the protection of proteins and membranes under various stresses (hyperosmotic, freezing, heat and oxidative stress) [72]. As shown recently by Freeman et al. [73], the deletion of orthologous operons in *Pseudomonas syringae *pv. *tomato *eliminated trehalose accumulation and reduced bacterial growth under hyperosmotic conditions. In addition, PA2144 (*glgP*), which encodes glycogen phosphorylase, may participate in the synthesis of trehalose precursors. Moreover, we observed increased expression of PA2416 (*treA*), situated aside from the PA2134-92 cluster, which codes for periplasmic trehalase precursor. The TreA homolog in *E. coli *is an osmotically inducible enzyme necessary for the catabolism of trehalose [74]. In addition, gene PA3757 coding for probable trehalose operon repressor was significantly downregulated in the mutant strain (5.59-fold) which could result in observed increased expression of other trehalose metabolism-related genes. Therefore, it is possible that increased expression of trehalose biosynthetic genes in the mutant strain could be responsible for the increased resistance of the mutant to the hyperosmotic stress, as we showed in the phenotypic analyses.

Among the other affected QS-regulated genes, many of which are also regulated by stationary phase σ-factor RpoS, were genes encoding virulence factors such as alkaline protease AprA and components of its secretion apparatus AprD and AprF, the *chiC *gene coding for chitinase, the elastase LasB gene, and *pa1L*, which encodes PA-I galactophilic lectin. The rest of the QS-controlled genes were either genes of primary metabolism or hypothetical genes.

#### Genes of the Pho regulon

Several genes associated with phosphate acquisition showed differential transcription in the mutant strain under conditions of oxidative stress (Table 5). The genes *pstS *and *phoU *of the phosphate-specific membrane transport complex PstSCAB-PhoU, *phoB*, the principal two-component response regulator [75], as well as some other genes of the Pho regulon were less upregulated in the mutant than in the wild-type response. Expression of the Pho regulon, under the conditions of inorganic phosphate (P_i_) limitation, is activated by the binding of PhoB to a consensus PHO box sequence within the promoters of Pho regulon genes [76]. Consequently, we identified affected genes with predicted PHO boxes encoding transcriptional regulators PsrA [77] and CifR [78], *aprA *gene, which codes for alkaline protease, and several hypothetical genes.

Pho regulon genes were shown to be induced in *P. aeruginosa *PAO1 after the exposure to human respiratory epithelia [71,79]. As shown by Jensen et al. [76], PhoB regulates the expression of the QS regulator RhlR and PHO box consensus sequences were found in the promoters of many virulence regulators and genes. Furthermore, PstS protein was found to be involved in adherence to and disruption of the integrity of cultured intestinal epithelial cell monolayers [80]. These findings suggest that tight regulation of phosphate acquisition within the host tissues through the functional transport/regulatory Pst-Pho complex could play an indispensable role in the virulence of *P. aeruginosa*.

In summary, our microarray analysis of the transcription profile of a *pppA-ppkA *mutant under conditions of oxidative stress revealed that the response of the mutant strain to the oxidative stress is less coordinated than that of the wild-type strain. For example, in the mutant strain iron metabolism genes are less downregulated. On the contrary, the oxidative stress-adaptation genes as well as the genes of SOS regulon are less upregulated. These alterations in the cellular response to stress conditions would result in greater extent of damage, as manifested by increased expression of pyocin genes, and above all, by a significantly reduced survival of the mutant strain grown under the wide range of hydrogen peroxide concentrations. Furthermore, in addition to the genes regulated directly or indirectly by the oxidative stress, we identified differentially regulated genes of QS regulon along with the closely connected RpoS regulon. Overexpressed locus PA2134-92, which probably encodes several genes involved in trehalose biosynthesis, could play a role in the increased resistance of the mutant strain to hyperosmotic stress. The Pho regulon genes, downregulated in the mutant strain, were shown to be upregulated in the human respiratory epithelia. This fact suggests that phosphate availability and the regulation of phosphate acquisition can influence the virulence of *P. aeruginosa*.

### Complementation restores expression of affected genes to the wild-type level

To exclude the possibility of either polar or suppressor mutations in the mutant, we constructed complementation strains with wt *pppA-ppkA *genes expressed ectopically. By using the mini-Tn7 vector, pUC18-mini-Tn7T-LAC, with a *tac *promoter, *pppA-ppkA *genes were inserted into the chromosome of Δ*pppA-ppkA *mutant creating Δ::tn7TLACpak strain. A modified wild-type strain, PAO1::tn7TLAC, and mutant strain, Δ::tn7TLAC, with an inserted empty mini-Tn7T-LAC cassette were used as controls (see Material and Methods).

**Figure 5:**
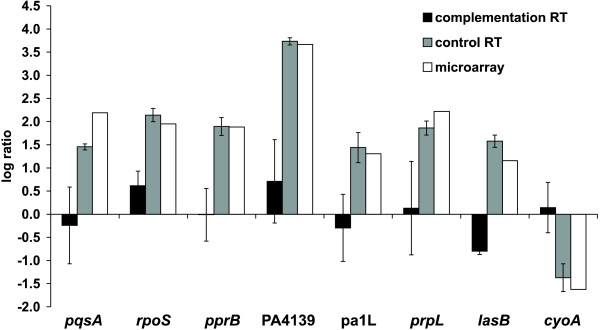
**Transcript levels of selected genes in the complemented mutant strain Δ::tn7TLACpak compared to the PAO1::tn7TLAC strain, and mutant Δ*pppA-ppkA *strain versus wild type as observed from microarray and control real-time PCR analysis**. For real-time PCR analysis, mean values of three biological replicates are given. Error bars represent the standard error of the mean. Complementation RT - transcript levels of the indicated genes in the Δ::tn7TLACpak compared to the PAO1::tn7TLAC strain assessed by real-time PCR; control RT - transcript levels of indicated genes in the Δ*pppA-ppkA *mutant compared to the PAO1 wild type assessed by real-time PCR; microarray - transcript levels of indicate genes in the Δ*pppA-ppkA *mutant compared to the PAO1 wild type assessed by microarray analysis.

By using quantitative real-time PCR analysis, we compared the expression levels of *pppA *and *ppkA *in strains PAO1::tn7TLAC and Δ::tn7TLACpak either uninduced or induced by 1 mM IPTG. Three independent replicates of total RNA from each strain were isolated from cultures grown in minimal M9 medium up to OD_600 _= 0.8. As shown in Table 6, the expression of *pppA *and *ppkA *in the Δ::tn7TLACpak increased approximately 30-fold upon induction with 1 mM IPTG when compared to the PAO1::tn7TLAC strain. Interestingly, *pppA *and *ppkA *were expressed in the Δ::tn7TLACpak strain even without induction with IPTG, and the transcript levels were comparable to those detected in the PAO1::tn7TLAC strain. Therefore, further qRT-PCR analysis of genes affected in the Δ*pppA-ppkA *mutant was performed with cultures without the presence of an inducer. The results showed that the expression of all selected genes in the complemented Δ::tn7TLACpak strain recovered approximately wild-type levels (Figure 5), and thus confirmed the role of PppA-PpkA in their regulation.

**Table 6:** Fold change of expression of *pppA *and *ppkA *genes in the strains PAO1::tn7TLAC, Δ::tn7TLAC and Δ::tn7TLACpak.

	Fold change^a^	
	
Gene	Δ::tn7TLACpak+IPTG/ PAO1::tn7TLAC+IPTG	Δ::tn7TLACpak/ PAO1::tn7TLAC
*pppA*	27.49 ± 5.36	-1.32 ± 0.34^b^
*ppkA*	35.16 ± 6.76	1.59 ± 0.67^b^

^a ^Fold changes represent the ratio of the expression levels in the comparison of *P. aeruginosa *Δ::tn7TLACpak or Δ::tn7TLAC, respectively, and PAO1::tn7TLAC. Minus (-) sign indicates a decreased expression in Δ strains;^b ^Fold change not significantly different from PAO1::tn7TLAC (*P *> 0.05); + IPTG - induction of expression with 1 mM IPTG

To confirm further complementation of the mutant strain we performed several phenotypic analyses. Growth analysis of the complemented strain Δ::tn7TLACpak in M9 minimal medium with glucose showed intermediate phenotype when compared to the PAO1::tn7TLAC and Δ::tn7TLAC strains. The complemented strain recovered approximately wild-type (PAO1::tn7TLAC) levels resistance to both oxidative and osmotic stress conditions. The Δ::tn7TLACpak strain significantly rescued the defect in pyoverdine production. In addition, the Δ::tn7TLACpak strain was able to induce necrosis of the lettuce leaves in the plant virulence model, although to the lesser extent than the PAO1::tn7TLAC strain. All figures documenting complemented mutant phenotypes are provided as Additional file 3.

## Conclusions

Our microarray analysis of the transcription profiles of a *pppA-ppkA *mutant revealed that the posttranslational modification of an as-yet-unidentified target(s) affects the expression of many functionally different genes in *P. aeruginosa*. The pleiotropic effect of the mutation is noteworthy. Among the most dramatically affected genes were those included in the cellular response to oxidative stress. The range of this adaptive response involving all affected genes was far inferior in the mutant when compared to the wild-type strain. Therefore, the increased susceptibility of the mutant to oxidative stress conditions could be a factor affecting survival in a cellular bactericidal system.

Due to the transmembrane topology of PpkA and the presence of an extracellular sensor domain containing a single von Willebrand factor A-domain (VWA domain), we hypothesised that PpkA could transmit environmental signals into the cell. Recent studies have demonstrated that ligand binding to potential extracellular sensor domains of bacterial PKs might be a prerequisite for kinase dimerisation and, consequently, for activation during an autophosphorylation process [81,82]. The phenomenon of ligand recognition-induced dimerisation of PpkA followed by autophosphorylation has been recently described [21]. In addition, it was showed that interaction of TagR, a likely ligand co-receptor, with C-terminal domain of PpkA can efficiently modulate its signal response. Therefore, it is tempting to speculate that under particular conditions PpkA could interact with other modulatory proteins resulting in altered substrate specificity.

PpkA has been recently shown to be a crucial regulatory element necessary for the recruitment of the newly identified type 6 secretion system and the secretion of Hcp1, VgrG1 and VgrG4 and Tse1-3 proteins [15,16,20]. The pleiotropic effect of the *pppA-ppkA *deletion on the cellular functions and physiology of *P. aeruginosa *suggests that this post-translational regulation of protein secretion is very likely only one of the molecular mechanisms that are under PpkA-PppA control. This hypothesis was proven by global analyses of transcriptomes of the wild-type and mutant strains under stress-free and oxidative stress conditions.

We hypothesise that deregulation of the response to environmental conditions such as oxidative stress and interference with RpoS/QS regulons as well as genes of the phosphate acquisition system would lead to decreased survival of the mutant within the host and attenuated virulence. These features were proven by the macrophage survival assay and in the plant model of infection. We suggest that the PppA-PpkA regulation pair, besides its role in T6SS, may also exert a direct or indirect effect on the regulation of stress responses likely through RpoS/QS regulons, thus influencing the virulence of *P. aeruginosa*. Therefore, it seems that PpkA targets some other substrate proteins outside H1-T6SS cluster and affects a broad range of cellular processes. It has been shown that other factors can contribute to *in vivo *substrate specificity of the individual kinases [83]. These factors are likely to include coordinated expression and colocalization of kinase and substrate, and protein-protein interactions. The identification of substrates and interacting factors that are involved in PpkA specificity will provide the grounds for further investigation.

## Authors' contributions

PB and JG designed the study. JG performed experiments, analysed data and drafted the manuscript. AU assisted with bioinformatic analysis and interpreting analysis results. KH built initial constructs and assisted in the construction of mutant strain. PB supervised the project and edited the manuscript. All authors discussed the results and implications and commented on the manuscript at all stages. All authors read and approved the final manuscript.

## Supplementary Material

Additional file 1 **Table S1**. Oligonucleotides used in this study. Click here for file

Additional file 2 **Figure S1. Characteristics of *P. aeruginosa *PAO1 wild-type and Δ*pppA-ppkA *strains**. (A) Growth curves in King's A and King's B medium. (B) Growth curves in Casamino Acid medium (CAA). This figure presents growth curves of *Pseudomonas aeruginosa *PAO1 wild-type and *ΔpppA-ppkA *strains in King's media containing glycerol and CAA medium. Click here for file

Additional file 3 **Figure S2. Phenotypic analysis of complemented strain Δ::tn7TLACpak. (A) Growth curve in minimal M9 medium. (B) Survival upon exposure to oxidative stress. (C) Survival upon exposure to osmotic stress. (D) Production of pyoverdine. (E) Plant (lettuce) infection assay with the *P. aeruginosa *PAO1::tn7TLAC (1), Δ::tn7TLAC (2), and Δ::tn7TLACpak (3) strains**. This figure presents phenotypic features of the complemented strain Δ::tn7TLACpak in comparison with wild-type strain PAO1::tn7TLAC and mutant Δ::tn7TLAC strain. Click here for file
