# Supplementary material for: A eukaryotic-type signalling system of Pseudomonas aeruginosa contributes to oxidative stress resistance, intracellular survival and virulence
Source: BMC Genomics. 2011 Aug 31;12:437. doi: 10.1186/1471-2164-12-437 (PMC3224232; doi:10.1186/1471-2164-12-437)
Supplement: Additional file 2 — Figure S1. Characteristics of P. aeruginosa PAO1 wild-type and ΔpppA-ppkA strains. (A) Growth curves in King's A and King's B medium. (B) Growth curves in Casamino Acid medium (CAA). This figure presents growth curves of Pseudomonas aeruginosa PAO1 wild-type and ΔpppA-ppkA strains in King's media containing glycerol and CAA medium. [file 1471-2164-12-437-S2.PDF]

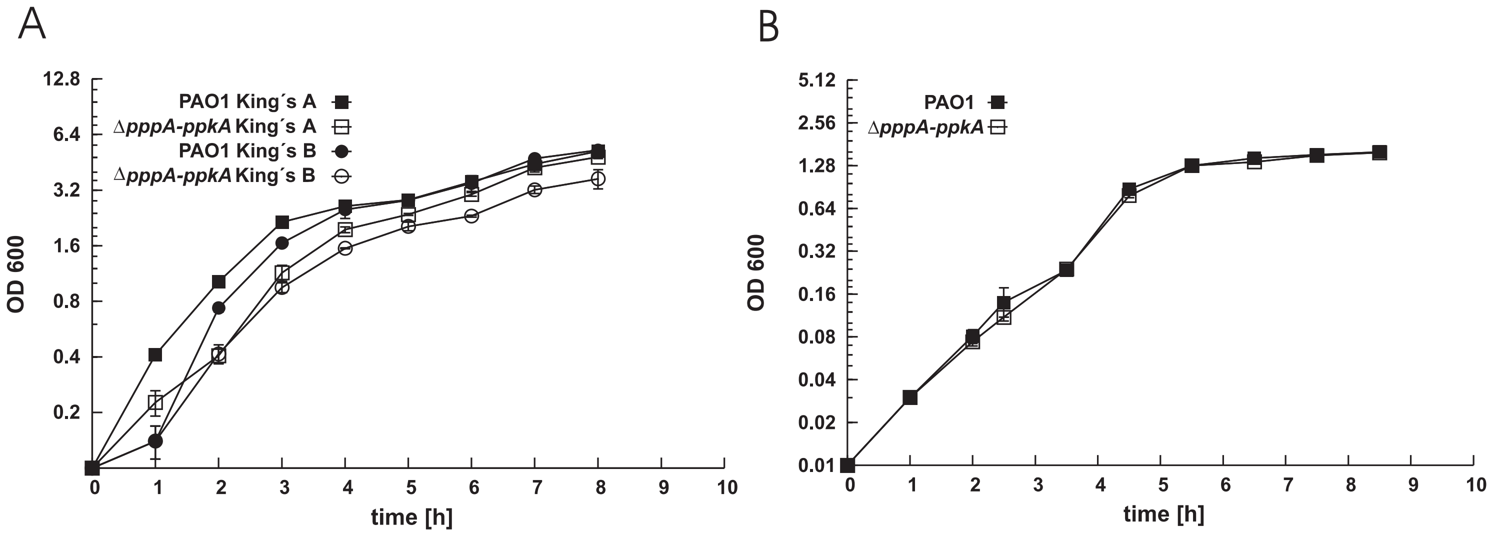

Figure S1. Characteristics of *P. aeruginosa* PAO1 wild-type and Δ*pppA-ppkA* strains. (A) Growth curves in King´s A and King´s B medium. (B) Growth curves in Casamino Acid medium (CAA). The standard errors of the means for three independent experiments are shown. Where error bars are not shown, the standard error was within the size of the symbol.
