# Supplementary material for: A eukaryotic-type signalling system of Pseudomonas aeruginosa contributes to oxidative stress resistance, intracellular survival and virulence
Source: BMC Genomics. 2011 Aug 31;12:437. doi: 10.1186/1471-2164-12-437 (PMC3224232; doi:10.1186/1471-2164-12-437)
Supplement: Additional file 3 — Figure S2. Phenotypic analysis of complemented strain Δ::tn7TLACpak. (A) Growth curve in minimal M9 medium. (B) Survival upon exposure to oxidative stress. (C) Survival upon exposure to osmotic stress. (D) Production of pyoverdine. (E) Plant (lettuce) infection assay with the P. aeruginosa PAO1::tn7TLAC (1), Δ::tn7TLAC (2), and Δ::tn7TLACpak (3) strains. This figure presents phenotypic features of the complemented strain Δ::tn7TLACpak in comparison with wild-type strain PAO1::tn7TLAC and mutant Δ::tn7TLAC strain. [file 1471-2164-12-437-S3.PDF]

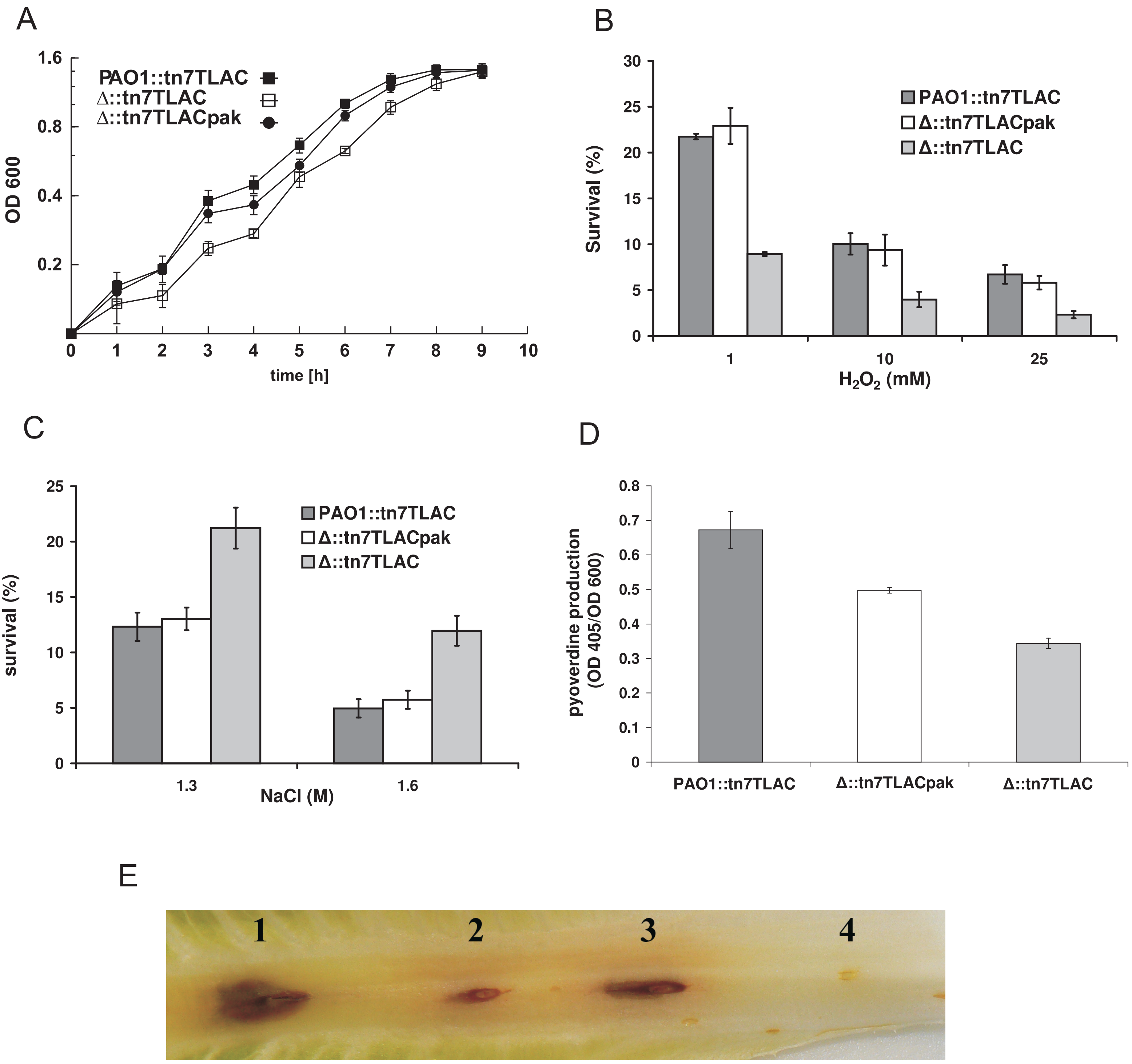

Figure S2. Phenotypic analysis of complemented strain Δ::tn7TLACpak. (A) Growth curve in minimal M9 medium. The standard errors of the means for three independent experiments are shown. Where error bars are not shown, the standard error was within the size of the symbol. **(B) Survival upon exposure to oxidative stress.** The standard errors of the means from three independent assays are shown. **(C) Survival upon exposure to osmotic stress.** The standard errors of the means from three independent assays are shown. **(D) Production of pyoverdine.** The standard errors of the means from three independent experiments are shown. **(E) Plant (lettuce) infection assay with the *P. aeruginosa* PAO1::tn7TLAC (1), Δ::tn7TLAC (2), and Δ::tn7TLACpak (3) strains.** Number 4 shows control (10 mM $MgSO_4$). The photograph shows a representative example of lettuce midribs after three days of infection.
